# Efficient metabolic fingerprinting profiling of extracellular vesicles for precise cancer diagnosis and treatment monitoring

**DOI:** 10.1016/j.mtbio.2025.101857

**Published:** 2025-05-12

**Authors:** Shurong Wang, Dongmei Liu, Ruoke Wang, Yan Zou, Tongtong Tian, Xuedong Huang, Xiaoni Fang, Baohong Liu

**Affiliations:** a Department of Chemistry, Shanghai Stomatological Hospital, School of Pharmacy, Institute of Biomedical Sciences, Fudan University, Shanghai, 200438, China; b Department of Pharmacy, Qingdao Municipal Hospital, Qingdao, 266001, China; c Department of Laboratory Medicine, Zhongshan Hospital, Fudan University, Shanghai, 200032, China

**Keywords:** EVs, Metabolites, LDI MS, AuM matrix, Cancer diagnosis and treatment monitoring

## Abstract

Breast cancer is the most prevalent cancer among women globally, underscoring the need for accurate prognostic and predictive biomarkers. Extracellular vesicles (EVs), carrying bioactive molecules such as proteins and miRNAs, have emerged as promising candidates for non-invasive liquid biopsy. However, their metabolic profiles remain underexplored. Here, we present a hybrid gold matrix (AuM), composed of gold nanospheres and nanorods, for EVs metabolome profiling via matrix-assisted laser desorption/ionization-time of flight mass spectrometry (MALDI-TOF MS). The AuM enhances ionization efficiency through its large surface area and anisotropic optical properties, enabling sensitive analysis with just 100 nL of serum and rapid processing time. Using this platform, we identified six potential metabolic biomarkers distinguishing early-stage breast cancer (BC) patients from healthy donors (HD). For treatment monitoring, serum-derived EVs from breast cancer mouse models treated with doxorubicin hydrochloride were analyzed, and a three-metabolite panel achieved 100 % accuracy in evaluating therapeutic response. This study reveals the metabolic heterogeneity of EVs and demonstrates the utility of EVs metabolomics in both diagnosis and treatment monitoring. Our findings bridge the gap between EVs-based laboratory research and clinical application, offering a promising tool for advancing precision medicine in breast cancer management.

## Graphical abstract

**Figure:**
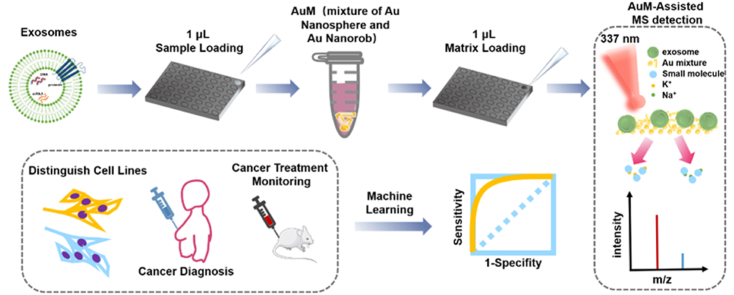

## 1 Introduction

Breast cancer （BC) is a disease in which abnormal breast cells grow out of control and form tumors [1]. In women worldwide, BC is the most common malignancy, and approximately 70–80 % of patients with early-stage non-metastatic disease can be cured [2]. Therefore, it is vital to find predictive biomarkers for the early diagnosis of BC, which is conducive to the early treatment of patients. Unlike predictive biomarkers, prognostic biomarkers help to monitor prognostic level [3]. It was reported that the existing BC treatment methods could make the survival rate of patients reach more than 90 % within 5 years, but these patients might also experience breast cancer recurrence, new primary breast cancer, other cancers, and short-term and long-term adverse reactions caused by treatment [4]. Consequently, prognostic biomarkers have the potential to predict the risk of disease recurrence, help clinician decision-making, evaluate drug efficacy, and facilitate individualized treatment, thus reducing mortality in complex diseases [[5], [6], [7]]. However, the low specificity and sensitivity of existing routine tests lead to insufficient early diagnosis or risk assessment, and the lack of symptoms in the prognostic phase makes later disease tracking difficult, so identifying useful predictive and prognostic biomarkers remains a great clinical challenge.

Extracellular vesicles (EVs), nanosized vesicles with diameters ranging from 30 to 150 nm enclosed within a phospholipid bilayer, are recognized as potent carriers for intercellular information and cargo transportation. These vesicles have been unequivocally shown to harbor vital information and cargoes, including proteins, miRNA, lipids, and other bioactive ingredients [[8], [9], [10]]. Such cargoes possess the ability to reflect the state of donor cells and function as biological markers for cell communication [11,12]. Furthermore, alterations in the mechanism governing EVs biogenesis, which relies on the endosomal sorting complexes required for transport (ESCRT), have been shown to give rise to EVs heterogeneity [10,13]. Distinct cell origins lead to varied proteins and metabolites sorting patterns in EVs, resulting in their intrinsic heterogeneity. This attribute of EVs provides a superior platform for unveiling origin-specific heterogeneity, consequently serving as a more accurate reflection of different disease stages, allowing for profound insights into disease pathways and facilitating timely clinical interventions [14,15]. To date, a considerable body of research has predominantly focused on the potential of EVs' proteins and miRNAs as promising biomarkers for cancer [[16], [17], [18]]. However, recent investigations have unveiled the significantly greater complexity of EVs’ cargoes, revealing the presence of diverse metabolites [10,19]. These metabolites have the capacity to be transferred through the EVs pathway to adjacent cancer cells, profoundly influencing the metabolic processes within recipient cells, and thus promoting the progression of cancer [20]. As a result, a group of metabolites or a single metabolite, whose concentration changes correlate with disease states, can be as predictive and prognostic biomarkers to participate in early diagnosis and monitor the status before and after drug treatment. In terms of clinical application, EVs can be sourced from liquid biopsies to capture the comprehensive systemic tumor status, in contrast to the limitations associated with traditional tissue biopsies obtained from singular or restricted spatial regions. Metabolites can meet the needs of non-invasive diagnosis and high-throughput detection. Therefore, the metabolic profiles of EVs from a variety of different sources effectively bridge the divide between conceptual validation and clinical applications, encompassing cancer cell characterization, cancer progression monitoring, and post-treatment evaluation.

Traditional approaches to metabolites (small molecules within molecular weight ≤1000 Da) analysis, typically employing liquid chromatography tandem mass spectrometry (LC-MS/MS), are distinguished by their exceptional capabilities in separation, reproducibility, resolution, and sensitivity [21]. Nonetheless, their utility in clinical diagnosis and prognosis is hindered by the intricate and time-consuming sample preparation procedures. In contrast, MALDI-TOF MS presents an array of advantages, including simplicity of operation, time-saving, high throughput, and robustness against contamination [22,23]. However, the traditional matrix of MALDI-TOF MS confronts two principal limitations when applied to metabolic analysis. Firstly, conventional organic matrices, exemplified by alpha-cyano-4-hydroxycinnamic acid (CHCA) and 2,5-dihydroxybenzoic acid (DHB), are fraught with substantial background interference owing to self-dissociation within the lower mass range, which corresponds to the mass range of metabolites [24]. Secondly, the conventional matrix is susceptible to uneven crystallization with stochastic factors, thereby constraining reproducibility and stability [25]. Therefore, the Au nanoparticles were introduced to ameliorate these inherent limitations of organic matrix in metabolism analysis. Characterized by their low heat capacity and high thermal conductivity, Au nanoparticles with a high yield of hot carriers can accelerate charge transfer at the analyte substrate interface for metabolic detection [26]. Within Au nanoparticles, the Au nanosphere ample surface area permits enhanced analyte adsorption and offers robust stability, alongside exceptional biocompatibility, rendering it as the primary candidate for biological sample analysis [27,28]. Furthermore, Au nanorod exhibits distinctive anisotropic properties, capable of generating unique longitudinal and transverse localized surface plasmon resonance (LSPR) modes, which prove advantageous in matrix applications [[29], [30], [31]].

**Fig. 1:**
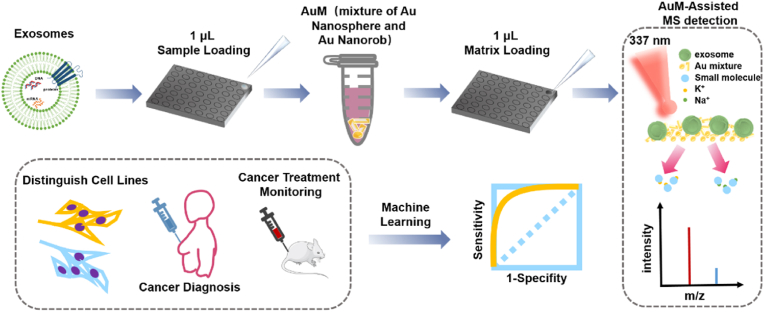
Schematic illustration of the distinctive-source EVs metabolic profiling on AuM-assisted LDI MS detection.

In this research, we proposed a hybrid Au matrix (AuM) composed of Au nanospheres and Au nanorods for the metabolic profiles of EVs using MALDI-TOF MS (AuM-assisted LDI MS), as depicted in Fig. 1. The AuM matrix leveraged the advantages of an expansive surface area and distinctive anisotropic properties, optimizing light absorption and ionization enhancement efficiency. Initially, we validated the efficacy of the AuM platform in distinguishing different categories of cancer cells. Subsequently, the approach was applied to analyze EVs-derived from the serum of BC patients and healthy donors (HD), revealing differences in metabolic profiles of EVs between these groups. This holds promise for identifying predictive biomarkers for BC. Notably, the use of only 100 nL of serum enabled differentiation between EVs originating from these two groups. By employing AuM-assisted LDI MS, we identified six potential predictive biomarkers (glucose, lactic acid, serine, 2-hydroxy-3-methylpentanoic acid, glycerophosphocholine, and histidine) that exhibited correlations with metabolic pathways such as glycolysis and choline metabolism, which are highly relevant to tumor initiation and migration. To comprehensively assess the effectiveness of BC drug treatment, we also utilized AuM-assisted LDI MS to analyze metabolic patterns of EVs in mice serum across different groups, including treatment and tumor groups. Through a search, we identified twenty distinct metabolites and screened three prognostic biomarkers (alanine, phosphocholine, and histidine) specifically associated with cancer to create a biomarker panel. This panel served as a valuable tool for monitoring the efficacy of cancer treatments, achieving an impressive accuracy rate of 100 %. By comparing metabolite profiles of EVs between the treatment and tumor groups, we observed patterns of changes in metabolites of EVs throughout treatment. The AuM platform demonstrated significant potential for metabolic profiling of EVs in cell distinction, cancer diagnosis, and treatment monitoring.

## 2 Experimental section

### 2.1 Chemicals and reagents

Au nanospheres (AuNS) were purchased from Jiangsu Xianfeng Nanomaterial Technology Co., Ltd (China). Dulbecco's modified essential medium (DMEM), roswell park memorial institute (RPMI) 1640 medium, phosphate buffered saline (PBS), phosphotungstic acid and ethylene diamine tetraacetic acid (EDTA) were ordered from Beijing Solarbio Science & Technology Co, Ltd. (China). The non- EVs fetal bovine serum (FBS) was purchased from Shanghai VivaCell Biosciences Co, Ltd. (China). 3,3′-dihexyloxacarbocyanine iodide (DiO dyes) was ordered from Sangon Biotech Co, Ltd (China). The syringe, sterile cell strainer (70 μm) and 0.22 μm filter were purchased from Titan Scientific Co, Ltd (China). The bicinchoninic acid (BCA) protein quantitative kit, trypsin, and penicillin-streptomycin were purchased from Thermo Fisher Scientific Inc (USA). The HeLa, Huh-7 and 4T1-Luc cell lines were obtained from American Type Culture Collection. The cetyltrimethylammonium bromide (CTAB), HAuCl_4_ (0.01 M), silver nitrate, hydroquinone, NaBH, α-Cyano-4-hydroxycinnamic acid (CHCA), ethanol, acetonitrile (ACN), trifluoroacetic acid (TFA), doxorubicin hydrochloride, sucrose and ultrafilter (3 kDa) were purchased from Sigma-Aldrich (USA). The deionized (DI) water used in all experiments was obtained from a Milli-Q system (Millipore, USA).

### 2.2 Isolation of EVs from cell medium

In this study, HeLa cells and Huh-7 cells were cultured under standard conditions. The cells were maintained in DMEM supplemented with 10 % FBS in a 5 % CO_2_ incubator at 37 °C. When the cells reached approximately 75 % confluence, they were washed three times with PBS to remove any residual media and cellular debris. Following the washes, the cells were replenished with non-EVs medium, which consisted of DMEM supplemented with 10 % FBS that had been depleted of EVs. After 48 h of incubation in the non-EVs medium, the culture supernatant was collected for EVs isolation.

The gold standard method of EVs isolation, known as differential centrifugation, was employed to isolate EVs from cell culture medium. The following steps were carried out at 4 °C to maintain the integrity of the EVs. The collected cell culture supernatant was first centrifuged for 10 min at 500 *g*, followed by 2000 *g* centrifugation for 20 min, and finally at 10,000 *g* centrifugation for 30 min. The above process was to remove cell debris and apoptotic body of non-EVs particles. The EVs were harvested by ultracentrifugation (at 100,000 *g*) for 70 min and washed with 1 mL of cold PBS. The final isolated EVs were resuspended in 100 μL of PBS and stored at −80 °C for further use in downstream analyses.

### 2.3 Isolation of EVs from human serum

Serum samples from BC patients were obtained from consented donors at RenJi Hospital, which has been approved for the use of human serum samples. 10 mL of peripheral blood was collected and placed at 4 °C for 4 h, and then centrifuged (at 2000 *g*) for 15 min at 20 °C. The supernatant was centrifuged again (at 10,000 *g*) for 30 min at 20 °C to remove cell debris and large vesicles. For EVs extraction, the prepared serum was diluted to 10 folds with PBS. The diluted serum was ultracentrifuged (at 100,000 *g*) for 70 min and washed with 1 mL of cold PBS. The EVs were resuspended into cold PBS, and stored until used at −80 °C.

### 2.4 Preparation of mouse model

To investigate the effects of tumor development and drug treatment, female BALB/c regular mice were used to build a mouse model. After one week of adaptive feeding, the mice were randomly divided into two groups (tumor-bearing group and drug-treatment group). The mice in the tumor group and drug-treatment group underwent the same pre-treatment process. In this pre-treatment process, each mouse was inoculated with tumor cell (4T1-Luc) suspension. Firstly, the 4T1-Luc cell lines were cultured in a 37 °C cell incubator with 5 % CO_2_. And the medium (RPMI 1640 medium with 10 % FBS) was changed every 2–3 days. Then the cells were taken in the logarithmic growth phase and the cell concentration was adjusted to 1 × 10^5^ cells per mL. After disinfection of mice with 75 % ethanol, each mouse was injected subcutaneously with 0.1 mL of 4T1-Luc cell suspension (containing 1 × 10^4^ cells).

Each mouse in the drug-treatment group received intraperitoneal injections of sterile doxorubicin hydrochloride solution every 3 days. The mice in the tumor-bearing group, on the other hand, were injected with an equivalent dose of normal saline. The sterile doxorubicin hydrochloride solution (0.5 mg/mL) was prepared using physiological saline and filtered through a 0.22 μm filter membrane.

### 2.5 Preparation of mouse serum

For the collection of blood samples from mice, capillaries with a diameter of 0.5 nm were broken into 1 cm pieces and pre-soaked in EDTA saturated solution. After one week of medication, blood was collected from the retroorbital plexus vein of the mice. The collected blood samples were placed at 4 °C for 4 h, followed by centrifugation at 2000 g for 10 min at 20 °C. Subsequently, another centrifugation step was performed at 3500 g for 10 min at 20 °C. To eliminate large vesicles, the supernatant was centrifuged again at 10,000 g for 30 min at 20 °C. The resulting supernatant was stored at −80 °C. The prepared mouse serum was diluted by a factor of 10 using PBS. The solution was then subjected to ultracentrifugation at 100,000 g for 70 min and washed with 1 mL of cold PBS. The pellet was resuspended in cold PBS and stored at −80 °C until further use.

### 2.6 Preparation of Au nanorobs (AuNR)

The synthesis of AuNR was carried out using the seed-mediated method. The growth solution consisted of 17.88 mL of CTAB solution (0.11 M) and 800 μL of HAuCl4 (0.01 M). Subsequently, 61 μL of silver nitrate (0.02 M) and 526 μL of hydroquinone (0.1 M) were added to the mixture and gently shaken. The solution was allowed to stand for 5 min at 30 °C. Then, 4 μL of NaBH (0.498 mM) was added, and the solution was left to stand for 12 h at 30 °C to allow the pellet to form. The pellet was collected by centrifugation at 10,277 g for 20 min. After two washes, the purified AuNR pellet was resuspended in CTAB solution (0.01 M) and stored in the dark at room temperature.

### 2.7 Characterization of Au nanomaterials

For the measurement of transmission electron microscope (TEM), the Au nanomaterials were resuspended in DI water, and with ultrasonic for better dispersion. The 5 μL of prepared solution was dropped on 200 mesh carbon-coating copper grids, and dehydrated in air. The samples were observed by the JEOL 2011 transmission electron microscope operated at 200 kV (JEOL, Japan). The Zeta potentials of Au nanomaterials were measured using Zeta cell by Zetasizer Nano (Malvern, England). UV–vis absorption spectra of AuNR, AuNS, and AuM were measured by UV–vis spectrophotometer, HP8453 (Agilent Technologies Inc., USA). The spectral range was set as 300–800 nm (1 nm resolution) using ultrapure water as a solvent blank.

### 2.8 Characterization of EVs

To quantify the EVs particles, the samples were characterized by nanoparticle tracking analysis (NTA) with 488 nm laser. Firstly, all samples were diluted with PBS buffer and filtered using a 0.22 μm filter membrane. Subsequently, the samples were analyzed at room temperature and measured three times at 25 frames per second. For fluorescent microscopy observation, the EVs were incubated with DiO dye at a 1:1 ratio at 37 °C in a dark environment for 30 min. The dyed EVs were then enriched using an ultrafiltration membrane (MW: 3 kDa) to remove excess dye. Fluorescence images of the EVs were captured using Total internal reflection fluorescence microscopy (Olympus, Japan) with a 480 nm laser. For TEM characterization of EVs, the samples were immobilized in a PBS solution containing 2.5 % glutaraldehyde for 5 min. They were then incubated on 200 mesh carbon-coated copper grids for 1 min and excess fluid was removed using filter paper. Next, the samples were negatively stained with 2 % phosphotungstic acid for 30 s and excess fluid was aspirated using filter paper. To achieve a clear background, the grids were washed twice with DI water and air-dried before measurement. The prepared samples were observed using a JEOL 2011 transmission electron microscope operated at 200 kV (JEOL, Japan).

### 2.9 MALDI-TOF MS detection

To perform the analysis, 1 μL of the detecting sample was dropped onto a Microflex Anchorchip plate (Bruker, Germany) and allowed to dry under ambient air. Subsequently, 1 μL of matrix solution was added to the dried sample. Additionally, CHCA (10 mg/mL in 50 % ACN/47.5 % water/2.5 % TFA, percentage by volume) was deposited onto the plate for mass calibration. The detecting sample was then analyzed using a Microflex LRF MALDI-TOF Mass Spectrometer (Bruker, Germany) equipped with a pulsed 337 nm nitrogen laser. The MALDI-TOF MS analysis was performed in the linear positive mode, with a mass scan range (*m*/*z*) of 100–1000. The optimized parameters were set as follows: 75 % laser intensity, laser attenuator with a 35 % offset and a 40 % range, accumulation of 1000 laser shots, 10.0 × detector gain, and a 120 ns delayed extraction time. Each sample was measured three times in parallel.

### 2.10 Data analysis

The raw data of MALDI-TOF MS was transferred into text format by Flexanalysis (Bruker, Bremen, Germany). The R packages of MALDIquant and MALDIquantForeign (http://www.strimmerlab.org/software/maldiquant/) (Gibb and Strimmer, 2012) were performed to align mass value after Savitzky-Golay smoothing and statistics-sensitive nonlinear iterative peak-clipping (SNIP) baseline correction. The parameters were set as follows: the signal-to-noise ratio of peak detection was 4, the half window size was 70 and peaks were removed with the binPeaks function with a tolerance of 0.005.

Statistical analysis was performed by Metaboanalyst 5.0 (https://www.metaboanalyst.ca) [32]. The principal component analysis (PCA), partial least squares-discriminant analysis (PLS-DA), hierarchical clustering, and univariate analysis were obtained by univariate statistical analysis. Receiver Operating Characteristic Curve (ROC) curves were obtained by biomarker analysis. Other figures were plotted by Origin software (OriginLab, Northampton, USA) and Prism GraphPad (GraphPad Software, Boston, USA). The metabolites information was searched from the Human Metabolome Database (HMDB) (https://hmdb.ca) [33].

## 3 Results and discussion

### 3.1 Characterization of Au nanomaterials

**Fig. 2:**
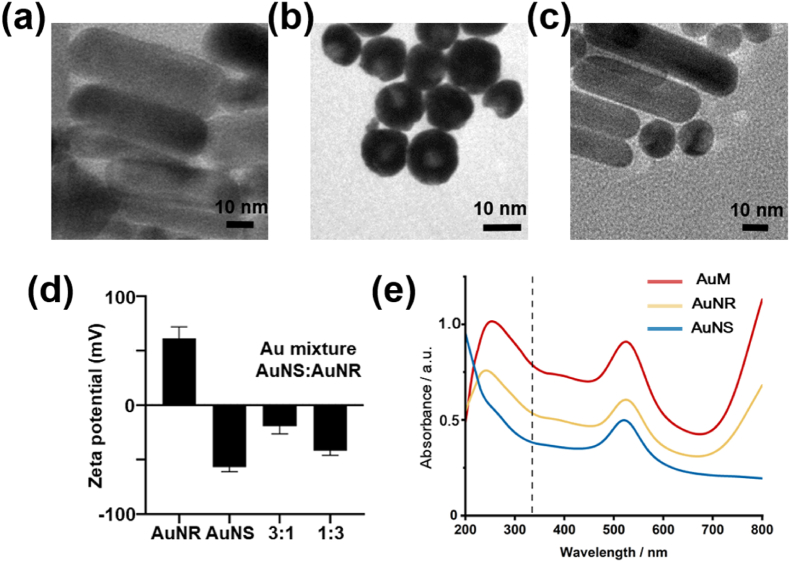
(a) The morphology characterization of Au nano-rod (AuNR), (b) Au nano-sphere (AuNS) and (c) mixture of AuNR and AuNS (AuM) by TEM measurement. (d) Zeta potential of AuNR, AuNS and AuM (AuNS:AuNR = 1:3 or 3:1 in volume). (e) The ultraviolet visible (UV–vis) absorption spectra of AuNR, AuNS and AuM (AuNS:AuNR = 1:3 in volume). The dashed line represented the laser wavelength of 337 nm. Error bars indicate standard deviation from triplicate experiments.

Combining the advantages of clear background and high capability to enlarge signal, Au nanoparticles were adopted to be matrix to amplify the MALDI-TOF MS signal of metabolites. Due to the large surface area of the Au nano-sphere (AuNS) and the anisotropy of the Au nano-rod (AuNR), a new Au mixture (AuM, ratio as AuNS:AuNR in volume) has been developed by combining these two advantages for EVs metabolic profiles. Firstly, the AuNS, AuNR, and AuM were characterized using TEM (Fig. 2a–c). The images showed that AuNS, AuNR, and AuM were well dispersed. The AuNR had a long length of 60 nm and a wide length of 20 nm, with uniform size distribution. The size distribution of AuNS was around 15 nm. The AuM nanoparticles exhibited three main size distributions and two different shapes, which potentially improved the gold matrix assistance ability. Additionally, the Zeta potential of the Au nanoparticles varied due to different synthesis methods (Fig. 2d). The AuNR had a positive Zeta potential, while AuNS had a negative Zeta potential. Interestingly, the AuM (AuNS:AuNR = 1:3 or 3:1 in volume) also exhibited a negative zeta potential, suggesting that the positively charged AuNR predominantly determine the net surface charge of the system. The negatively charged substrate surface promoted the formation of a metal cation layer, facilitating the binding of metabolites and metal cations in the positive mode of MALDI-TOF MS. The AuM (AuNS:AuNR = 1:3 in volume) appeared to be more stable than AuM (AuNS:AuNR = 3:1 in volume) based on the lower Zeta potential.

The UV–vis absorption spectra of AuNR, AuNS, and AuM (AuNS:AuNR = 1:3 in volume) were measured. Although the LSPR bands vary in accordance with the Au particle size and shape, the all three AuNR, AuNS, and AuM showed sharp plasmon bands around 520 nm (Fig. 2e), which can be attributed to the synergistic effect from different factors. The results confirmed that Au nanoparticles possessed a strong LSPR effect, enabling them to efficiently collect laser energy. Among AuNR, AuNS, and AuM, AuM exhibited the highest absorbance at the laser wavelength of 337 nm, indicating its superior ability to absorb and transfer UV laser energy during LDI (laser desorption/ionization). When AuM was employed as the matrix of MALDI-TOF-MS, which was exposed to a UV light with a wavelength 337 nm, the electrons in the valence band (VB) of the semiconductor were elevated to the conduction band (CB) and then transferred to the Fermi level (EF) of the noble metal, leading to the separation of e^−^–h^+^ pairs [34]. On the other hand, the electromagnetic field of incident light can couple with the oscillation of electrons in the plasmonic metal, resulting in a strong enhancement of the local electromagnetic field near the surface of AuM [35]. The excited AuM act as concentrators and increase the local light intensity, accelerating the formation of e^−^–h^+^ pairs in the semiconductor nanostructure [36]. Notably, the hybrid AuM matrix was prepared using ultrasonic treatment (10 min, 40 kHz) and optimized mixing conditions, including overnight rotation at 4 °C. These conditions provided the most uniform distribution and reproducible performance among all tested protocols (Fig. S1). While some heterogeneity remains visible in the TEM images, it did not significantly impact assay reproducibility or signal enhancement. As a result, the LSPR mode in AuM can positively influence the matrix properties in MALDI-TOF MS applications by enhancing spectral intensity, improving sensitivity, enabling selective absorption, stabilizing the matrix, and increasing reproducibility of the analysis, ultimately enhancing the performance of MALDI-TOF MS in metabolic profiling of EVs.

### 3.2 The feasibility of metabolic profiling of EVs by AuM-assisted LDI MS

**Fig. 3:**
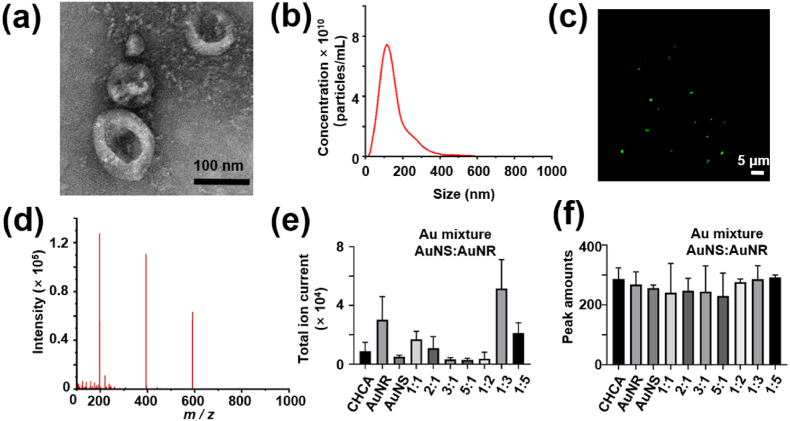
(a) The TEM image and (b) NTA measurement of the EVs model sample. (c) Fluorescence map and (d) typical mass spectra of EVs model sample. (e) The total ion current and (f) peak amounts of EVs mass spectra based on different matrices. AuNS, AuNR and AuM represented Au nano-sphere, Au nano-robs and the mixture of Au nano-sphere and Au nano-robs (ratio as AuNS: AuNR in volume), respectively. Error bars indicate standard deviation from triplicate experiments.

To comprehensively identify metabolic patterns of EVs using MALDI-TOF MS, an EVs model sample was prepared from HeLa cell lines through ultracentrifugation. The TEM image of the EVs model sample (Fig. 3a) revealed the distinct structure of vesicles, with bowl shapes and double layers. Additionally, the size distribution of the EVs model sample was measured using NTA (Fig. 3b). The peak concentration and peak size of the EVs model sample were determined as 7.45 × 10^10^ and 115 nm, respectively. The purity of the EVs was calculated by comparing the number of EVs particles to the amount of protein, which demonstrated high purity at 1.57 × 10^11^ (Fig. S2). The presence of bilayer lipids in the EVs model sample was confirmed through fluorescent imaging (Fig. 3c). From the above evidence, it can be concluded that the EVs model sample exhibited typical EVs characteristics. This sample can serve as a standard for evaluating the performance of EVs metabolic profiling using MALDI-TOF MS based on the AuM matrix (AuM-assisted LDI MS).

The raw spectrum obtained from AuM-assisted LDI MS is depicted in Fig. 3d, and some significant *m*/*z* values are listed in Table S1. A comparison was made between the peak amount and intensity achieved using the AuM matrix and the conventional organic matrix CHCA. Here, the optimized concentration of CHCA matrix was 10 mg/mL (Fig. S3). It was observed that the total ion current was significantly higher when using AuM matrix compared to CHCA (Fig. S4). The background signals of both matrices were also examined (Fig. S5). Unfortunately, the intensity of the CHCA background was even higher than that of the EVs model sample, which could be attributed to the high salt content in the sample. In contrast, the total ion current (TIC) of EVs based on AuM-LDI MS appeared to be less affected, and the AuM background exhibited less interference. This could be attributed to the high salt tolerance of the AuM matrix and its difficulty in ionization. Also, the AuM matrix highlighted excellent reproducibility (Fig. S6). These results confirmed the feasibility of EVs metabolic profiling using AuM-assisted LDI MS.

To optimize the experimental conditions for selective enrichment of EVs’ metabolites, different ratios of Au nanoparticles, including CHCA, AuNR, commercial AuNS, and various combinations of AuNR and AuNS, were employed. The goal was to amplify the MS signals of EVs. Comparing the results with CHCA, the mixture of AuNR and commercial AuNS exhibited nearly three times the signal enhancement in terms of the total ion current (TIC) (Fig. 3e). Additionally, the TIC obtained using the AuM matrix (a mixture of AuNS and AuNR) was significantly higher than that obtained using individual AuNS or AuNR. The total peak amounts identified using different matrices were comparable, with slightly more peak amounts observed with the AuM matrix (Fig. 3f). Consequently, the optimal ratio of AuNS to AuNR (1:3) was selected for subsequent experiments.

### 3.3 Discrimination of EVs metabolic profiles from different cells origins

**Fig. 4:**
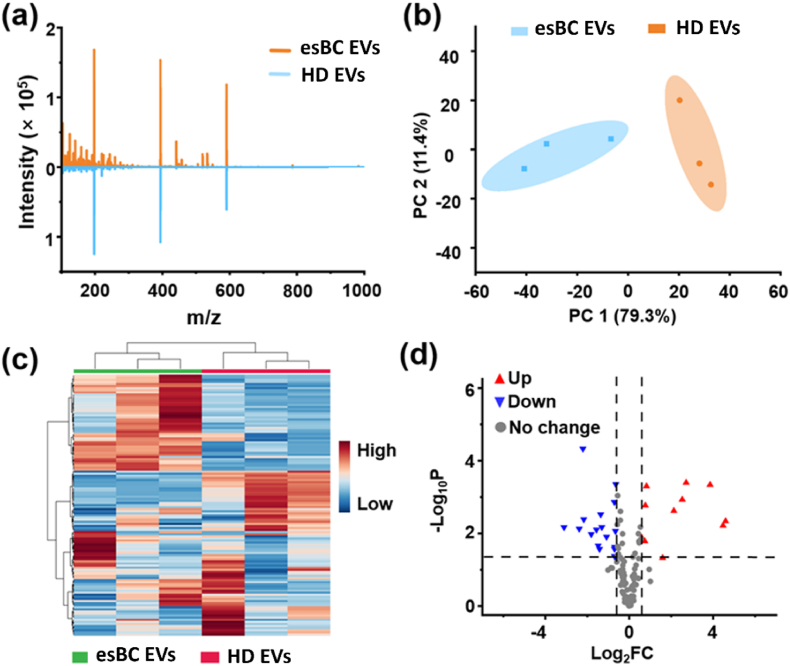
(a) Metabolic profiles of Huh-7 EVs and HeLa EVs by AuM-assisted LDI MS. (b) The PCA classification and (c) the heatmap of Huh-7 EVs and HeLa EVs based on AuM-assisted LDI MS data. (d) The volcano plot of differential small molecules between Huh-7 EVs and HeLa EVs. Red represented up-regulated molecules, and blue represented down-regulated molecules in Huh-7 EVs. Huh-7 EVs and HeLa EVs were isolated from Huh-7 cell lines and HeLa cell lines by ultracentrifugation, respectively. Three samples of Huh-7 EVs or HeLa EVs were measured, and AuM-assisted LDI MS detection of each sample was with three replicates. (For interpretation of the references to color in this figure legend, the reader is referred to the Web version of this article.)

To investigate the unique ability of AuM-assisted LDI MS in distinguishing the heterogeneity of EVs origins, we conducted an experiment using EVs from Huh-7 and HeLa cell lines, which represent liver cell-derived cancer cells and cervical cancer cells, respectively (Huh-7 EVs and HeLa EVs). As the result in Fig. 4a, the peak distribution of EVs metabolic profiles from Huh-7 EVs was different from HeLa EVs, which mainly displayed at 400–600 *m*/*z*. Meanwhile, the peak intensity of EVs metabolic profiles from HeLa EVs was less at 100–300 *m*/*z* and 450–580 *m*/*z*. To visualize these differences and effectively distinguish the metabolic patterns of EVs from the two cell origins, we employed principal component analysis (PCA). PCA, a non-supervised machine learning method, is commonly used to reduce the dimensionality of high-dimensional data. The metabolic profiles of EVs obtained through AuM-assisted LDI MS contained nearly 300 feature peaks, representing high-dimensional data. After conducting PCA analysis, the large dataset was simplified and represented in two main dimensions (PC1 and PC2), effectively discriminating the metabolic profiles of EVs from different cell lines (Fig. 4b). To further visualize the distinction between the metabolic patterns of EVs derived from different cell origins, we utilized an unsupervised hierarchical clustering heatmap. This heatmap visualized and separated the MS data of HeLa EVs and Huh-7 EVs, while also clustering the MS data of the same origin together (Fig. 4c). In Fig. 4d, the volcano plot displayed 11 up-regulated metabolites and 18 down-regulated metabolites identified in the Huh-7 EVs group compared to HeLa EVs (fold change (FC) > 1.5 and p-value (P) < 0.05). These significant features have the potential to differentiate EVs groups from distinct sources, representing differences in cell origins and their cargo. Overall, these findings demonstrate the unique ability of AuM-assisted LDI MS to distinguish the heterogeneity of EVs origins based on their metabolic profiles. This approach holds promise for applications in characterizing EVs in various diseases and identifying specific biomarkers.

### 3.4 EVs metabolic profiling discrimination of application on cancer diagnosis

**Fig. 5:**
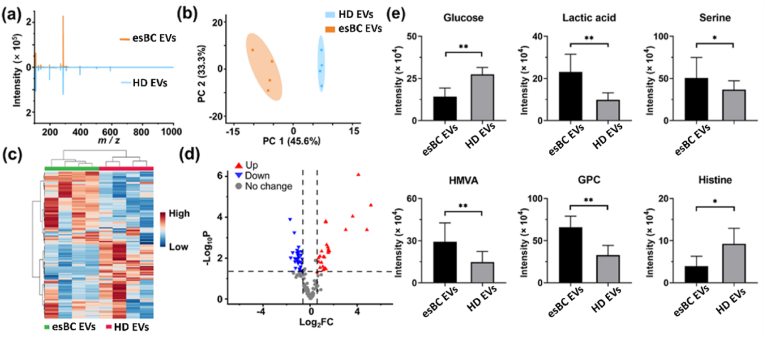
(a) AuM-assisted LDI-TOF mass spectra of esBC-EVs and HD-EVs. (b) The PCA classification and (c) the heatmap of esBC-EVs and HD-EVs based on AuM-assisted LDI MS data. The relative concentration corresponded to different groups listed in the right color box (red represented high concentration, blue represented low concentration). (d) The volcano plot of differential small molecules between esBC-EVs and HD-EVs. Red represented up-regulated molecules, and blue represented down-regulated molecules in esBC-EVs. “esBC-EVs” and “HD-EVs” mean EVs isolated from serum of early-stage BC patients and HD by ultracentrifugation, respectively. (e) Six key metabolite concentration difference in esBC-EVs and HD-EVs. The term “HMVA” referred to 2-hydroxy-3-methylpentanoic acid, and GPC meant glycerophosphocholine. ∗ and ∗∗ represented p < 0.05 and p < 0.01, respectively. P-value was calculated by *t*-test. Four EVs samples separated from early-stage BC samples and HD were measured, and the AuM-assisted LDI MS detection of each sample was performed with three replicates. (For interpretation of the references to color in this figure legend, the reader is referred to the Web version of this article.)

Given the potential of AuM-assisted LDI MS in distinguishing EVs from different cell origins, it is promising to utilize this method for the early diagnosis of BC. In this study, the EVs’ metabolic profiles of early-stage breast cancer patients (esBC- EVs) and healthy donors (HD-EVs) were investigated using AuM-assisted LDI MS. Fig. 5a clearly demonstrates that the esBC-EVs metabolic profiles exhibited different peak distribution from HD-EVs. Additionally, the difference about metabolic profiles between esBC-EVs and HD-EVs was able to be visualized and well distinguished by our method and PCA analysis. As depicted in Fig. 5b, the two clusters representing esBC-EVs and HD-EVs were well separated from each other, indicating a clear distinction between the metabolic patterns of EVs from BC patients and HD. This distinction is further supported by the heatmap analysis shown in Fig. 5c, where the features of esBC-EVs and HD-EVs exhibit different distributions. The heatmap highlights the variations in metabolic profiles between the two groups and reinforces the potential of using AuM-assisted LDI MS to differentiate EVs derived from BC patients and HD.

The above findings suggest that AuM-assisted LDI MS holds promise for the early diagnosis of BC by analyzing the metabolic profiles of EVs. By identifying specific metabolic patterns associated with BC, this approach may contribute to the development of non-invasive diagnostic methods for detecting BC at an early stage. Thus, the volcano plot analysis of metabolic patterns from esBC-EVs and HD-EVs was performed to find possible BC predictive biomarkers. As highlighted in Fig. 5d, 58 significant metabolites (26 up-regulated and 32 down-regulated) under the criteria that fold change (FC) > 1.5 and p-value (*P*) < 0.05 between esBC-EVs and HD-EVs were identified. Under the consideration that glucose metabolism is one of the important metabolic pathways in cancer metabolism, we focused on several metabolites related to glucose metabolism [37]. As shown in Fig. 5e, lactic acid and serine increased significantly in esBC-EVs, while glucose decreased significantly. This result might clarify that BC cells had a high demand for glucose uptake, and glucose might be used preferentially. In the process of anoxic or aerobic glucose metabolism in breast cancer cells, pyruvate (the final product of glucose metabolism) might be induced to produce more lactic acid, resulting in the acidification of the tumor microenvironment [38,39]. The acidic environment might promote cancer cell adhesion, migration, and invasion. In addition, the serine/glycine metabolic pathway was also related to glycolysis [40]. Some studies reported that the protein related to the serine metabolic pathway was highly expressed in triple-negative breast cancer (TNBC) [41], which was consistent with up-regulation of serine in esBC- EVs. Besides glucose, branched-chain amino acid (BCAA) is also an essential nutrient as an energy source for cancer growth [42]. The up-regulation of 2-hydroxy-3-methylpentanoic acid, which belongs to BCAA metabolism, showed that BCAA might be used by BC cells likely to provide carbon for gluconeogenesis. Furthermore, the choline metabolism represents a significant part of the tumor metabolome, which could be verified by the increasing level of glycerophosphocholine (GPC) in esBC-EVs [43]. The possible pathway in BC cells is shown in Fig. S7. While searching for key metabolites, we also noticed a significant down-regulation of histidine, *i.e.*, the main substrate for histamine production. As histamine is closely linked to cancer development and with elevated levels observed in a variety of human malignancies [44], the results suggested that breast cancer cells accelerated the use of histamine, thus causing the decline of histidine. To confirm the reliability of identified potential metabolites serve as biomarkers for BC diagnosis, the results were compared with Fourier Transform Ion Cyclotron Resonance mass spectrometry (FT-ICR-MS). As shown in Table S2, the six potential biomarkers detected by the FT-ICR-MS shows satisfied absolute molecular weight deviations under 180 ppm. It can be concluded that the above six metabolites have the potential to serve as biomarkers for breast cancer diagnosis.

### 3.5 EVs metabolic profiling discrimination of application on cancer treatment monitoring

**Fig. 6:**
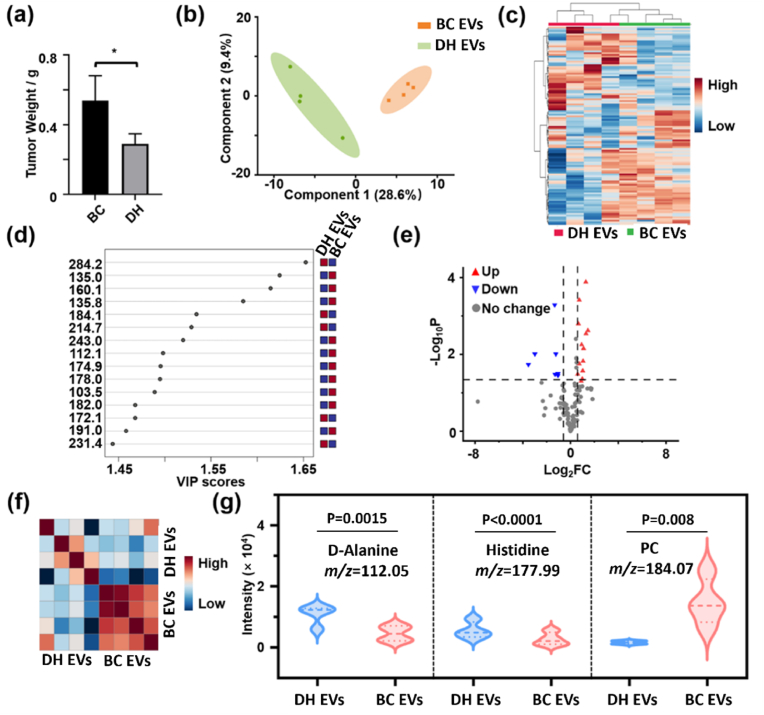
(a) The tumor weight of mice with breast cancer (BC) and doxorubicin hydrochloride treatment (DH). “∗” represented p value < 0.05. Error bars indicate standard deviation from triplicate experiments. (b) The PLS-DA classification and (c) top 25 features in the heatmap of BC-EVs and DH-EVs based on AuM-assisted LDI MS data. (d) Important AuM-assisted LDI MS features of BC-EVs and DH-EVs. Red and blue blocks represented high concentration and low concentration, respectively. (e) The volcano plot analysis of metabolic patterns from BC-EVs and DH-EVs. Red represented up-regulated, and blue represented down-regulated in DH-EVs. P-value was calculated by a *t*-test. (f) The co-relationship heatmap of metabolic patterns from BC-EVs and DH-EVs. (g) The mass spectra intensity of key features in BC-EVs and DH-EVs. PC means phosphatidylcholine. BC-EVs and DH-EVs represented serum-derived EVs from mice with BC and doxorubicin hydrochloride treatment, respectively. Four samples of EVs from mice with BC or doxorubicin hydrochloride treatment were measured, and AuM-assisted LDI MS detection of each sample was with three replicates. (For interpretation of the references to color in this figure legend, the reader is referred to the Web version of this article.)

To further expand the application of AuM-assisted LDI MS in treatment monitoring, we investigated the feasibility of monitoring cancer treatment through the discrimination of serum-derived EVs metabolic profiles. For this purpose, a mouse model was established with two groups: BC and doxorubicin hydrochloride treatment. EVs were isolated from mouse serum using differential ultracentrifugation. After the experiment, the tumor tissues were completely removed and weighed. As shown in Fig. 6a, the tumor weight in the treatment group was significantly lower than in the tumor group, indicating the successful establishment of the animal model. EVs were isolated from the BC and doxorubicin hydrochloride treatment groups (BC-EVs and DH-EVs), and the raw spectra were obtained using AuM-assisted LDI MS (Fig. S8). The EVs metabolic profiles of DH-EVs and BC-EVs exhibited differences in the number and intensity of peaks within the *m*/*z* 100–300 range, which could potentially serve as biomarkers. To identify signature peaks, Partial Least Squares Discriminant Analysis (PLS-DA) was employed to discriminate between the groups and select biomarker features. PLS-DA is a multivariate statistical analysis method used for supervised discriminant analysis. Unlike PCA, PLS-DA takes into account the impact of characteristic peaks on the classification and discrimination of each sample group through the variable importance for the projection (VIP). Thus, VIP scores obtained from PLS-DA can be used to identify potential biomarkers. As illustrated in Fig. 6b, the BC-EVs and DH-EVs groups were well separated after the application of PLS-DA. Furthermore, the unsupervised hierarchical clustering heatmap accurately clustered EVs samples from different groups (BC and DH) into distinct subgroups (Fig. 6c). The data from different groups exhibited distinct feature intensity distributions and were clearly separated from one another. Fig. 6d presents the top 15 *m*/*z* features based on their VIP scores, which can be utilized to construct a biomarker panel.

To further explore potential biomarkers that could serve as prognostic indicators, a volcano plot analysis was conducted on the metabolic patterns of BC-EVs and DH- EVs, as shown in Fig. 6e. The co-relationship heatmap also demonstrated that the metabolic profiles of BC-EVs and DH-EVs could be distinguished through AuM-assisted LDI MS (Fig. 6f). Interestingly, the DH-EVs group displayed greater heterogeneity in metabolic profiles compared to the BC-EVs group. This variation may arise from differences in individual treatment responses, such as variability in tumor regression, which could modulate EVs cargo composition. In Fig. 6e, a total of 24 significant metabolites were identified with fold change (FC) > 1.5 and p-value (P) < 0.05. Among these, 13 metabolites were up-regulated, while 11 metabolites were down-regulated in DH-EVs compared to BC- EVs (listed in Table S3). Similarly, the 24 potential biomarkers for BC treatment monitoring also show satisfied absolute molecular weight deviations under 180 ppm when FT-ICR-MS was used. By matching 20 key features with specific metabolite structures using the Human Metabolome Database (HMDB), pathway analysis was performed (Fig. S9). The pathway analysis revealed 26 metabolic pathways, including the tricarboxylic acid (TCA) cycle, glycine, serine, and threonine metabolism, phenylalanine, tyrosine, and tryptophan biosynthesis, histidine metabolism, glycolysis/gluconeogenesis, cysteine and methionine metabolism, among others (listed in Table S4). Some lipid metabolism pathways, such as glycerophospholipid metabolism, glycerolipid metabolism, and sphingolipid metabolism, were also enriched. These pathways are not only associated with cancer but also related to EVs budding. The enrichment of these pathways further supports the reliability of the discovered biomarkers. Next, more attention was given to the metabolites associated with the diagnostic markers mentioned above. Among 20 specific biomarkers, three breast cancer-related biomarkers (D-Alanine, *m*/*z* 112.05; Histidine, *m*/*z* 177.99; Phosphorylcholine, *m*/*z* 184.07) were selected to be further analyzed. As shown in Fig. 6g, they were significantly different in DH-EVs and BC- EVs. The decline of phosphorylcholine (PC) in DH-EVs might reveal that the tumor invasion of breast cancer was through total choline-containing compounds participated with phospholipid metabolism, which was consistent with the diagnostic biomarker (GPC) in Fig. S4b [45]. Moreover, the up-regulation of D-Alanine in DH-EVs might confirm that D-Alanine inhibits the invasiveness of malignant breast epithelial cells by altering metabolism and cellular acidity in vitro [46]. It was also found that histidine levels as one of the diagnostic markers rose in DH-EVs. This result showed that the histidine degradation pathway significantly affects the sensitivity of cancer cells to drugs, thereby affecting the therapeutic effect [47]. Consequently, the above three metabolites (D-Alanine, Histidine and PC) might construct the prognostic biomarker panel of BC to monitor medication levels. The receiver operating characteristic (ROC) curve was used to show the ability of a biomarker panel to distinguish between different groups, with a greater area under the curve (AUC) signifying enhanced discriminatory capacity. The AUC value of the classification ROC curve for the disease detection panel screened by our method was 1 (Fig. S10a). However, the AUC value of the ROC curves was 0.96 by using the top 5 features of conventional machine learning method (PLS-DA) for classification (Fig. S10b). To attain the AUC value of 1, it became necessary to expand the number of features to 50, a step that significantly prolonged data processing time. Therefore, our biomarker panel with less number and higher AUC value presented its strong prognostic utility. This result also confirmed that these three characteristic metabolites could be used as potential markers for large-scale clinical BC prognosis monitoring in the future.

## 4 Conclusions

In conclusion, the developed hybrid Au matrix coupled with MALDI-TOF MS analysis provides a rapid and high-throughput method for investigating EVs origin heterogeneity and metabolic profiling. By leveraging the unique properties of two Au matrices, this approach enhances LDI MS mass spectrometry analysis through efficient optical absorption and ionization, making it a valuable tool for discriminating EVs from different sources. The metabolomics analysis of EVs revealed that different cell types possess unique EVs subtypes, highlighting the heterogeneity of EVs. Additionally, six potential predictive biomarkers (glucose, lactic acid, serine, 2-hydroxy-3-methylpentanoic acid, glycerophosphocholine, and histidine) were identified, providing essential resources for the precise diagnosis of BC. These biomarkers also shed light on the pathways involved in tumorigenesis and metastasis. Furthermore, AuM-assisted LDI MS demonstrated its potential for monitoring the effectiveness of cancer treatment. It achieved a remarkable 100 % accuracy rate in distinguishing between the treatment and cancer groups in mice. This breakthrough in treatment monitoring offers a reliable and non-invasive approach for assessing the response to cancer therapy. Overall, AuM-assisted LDI MS overcomes the limitations of traditional diagnostic techniques and provides a more dependable avenue for early BC diagnosis and prognosis assessment. With its ability to investigate heterogeneity and metabolic profiling of EVs, this approach holds promise for advancing our understanding of cancer biology and improving patient outcomes. However, metabolite matching based only on the *m*/*z* values in its primary spectra has a considerable degree of error and uncertainty. Further refinements in nanoparticle formulation and assembly may help reduce variability in future studies. In addition, more accurate *m*/*z* values of metabolite characteristics obtained by high-resolution mass analyzer and background excluded could be combined to identify disease metabolic biomarkers. Moreover.

## CRediT authorship contribution statement

**Shurong Wang:** Writing – original draft. **Dongmei Liu:** Methodology. **Ruoke Wang:** Data curation. **Yan Zou:** Data curation. **Tongtong Tian:** Resources. **Xuedong Huang:** Data curation. **Xiaoni Fang:** Supervision. **Baohong Liu:** Funding acquisition.

## Declaration of competing interest

The authors declare that they have no known competing financial interests or personal relationships that could have appeared to influence the work reported in this paper.

## Data Availability

Data will be made available on request.
