# Supplementary material for: Efficient metabolic fingerprinting profiling of extracellular vesicles for precise cancer diagnosis and treatment monitoring
Source: Mater Today Bio. 2025 May 12;32:101857. doi: 10.1016/j.mtbio.2025.101857 (PMC12141543; doi:10.1016/j.mtbio.2025.101857)
Supplement: Multimedia component 1 [file mmc1.docx]

**Supporting Information**

**Efficient Metabolic Fingerprinting Profiling of Extracellular Vesicles for Precise Cancer Diagnosis and Treatment Monitoring**

Shurong Wang,^a^ Dongmei Liu,^b^ Ruoke Wang,^a^ Yan Zou,^a^ Tongtong Tian,^c^ Xuedong Huang,^a^ Xiaoni Fang,^a*^ Baohong Liu^a*^

a. Department of Chemistry, Shanghai Stomatological Hospital, School of Pharmacy, Institute of Biomedical Sciences, Fudan University, Shanghai, 200438, China

b. Department of Pharmacy, Qingdao Municipal Hospital, Qingdao 266001, China

c. Department of Laboratory Medicine, Zhongshan Hospital, Fudan University, Shanghai, 200032, China


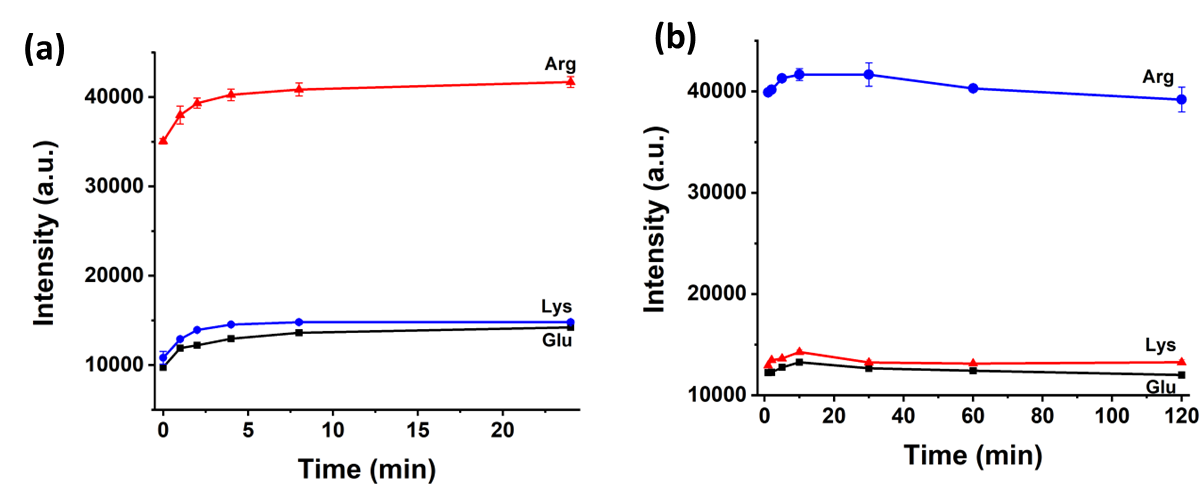


**Fig. S1.** Optimization of ultrasonic treatment and mixing durations (ranging from 1 hour to overnight gentle rotation) for the performance of AuM in terms of signal enhancement and reproducibility for 1mM amino acid standards (Arg, Lys, Glu) detection. Error bars indicate standard deviation from triplicate experiments.


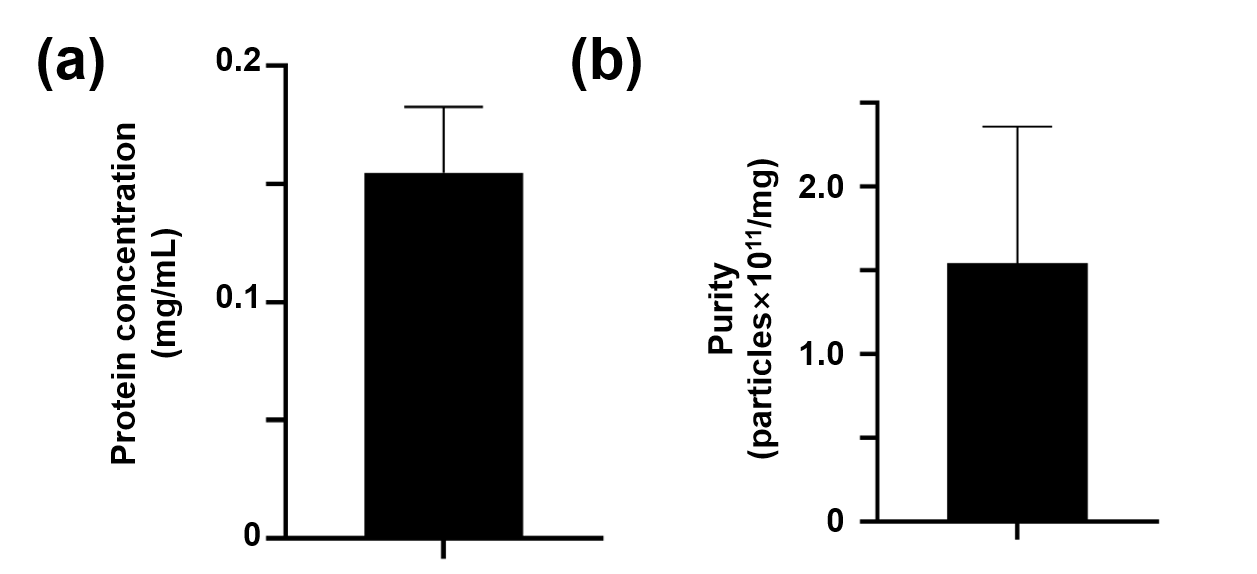


**Fig. S2**. (a) The protein concentration and (b) purity of EVs model sample. Purity was calculated by the number of EV particles over protein concentration. Error bars indicate standard deviation from triplicate experiments.


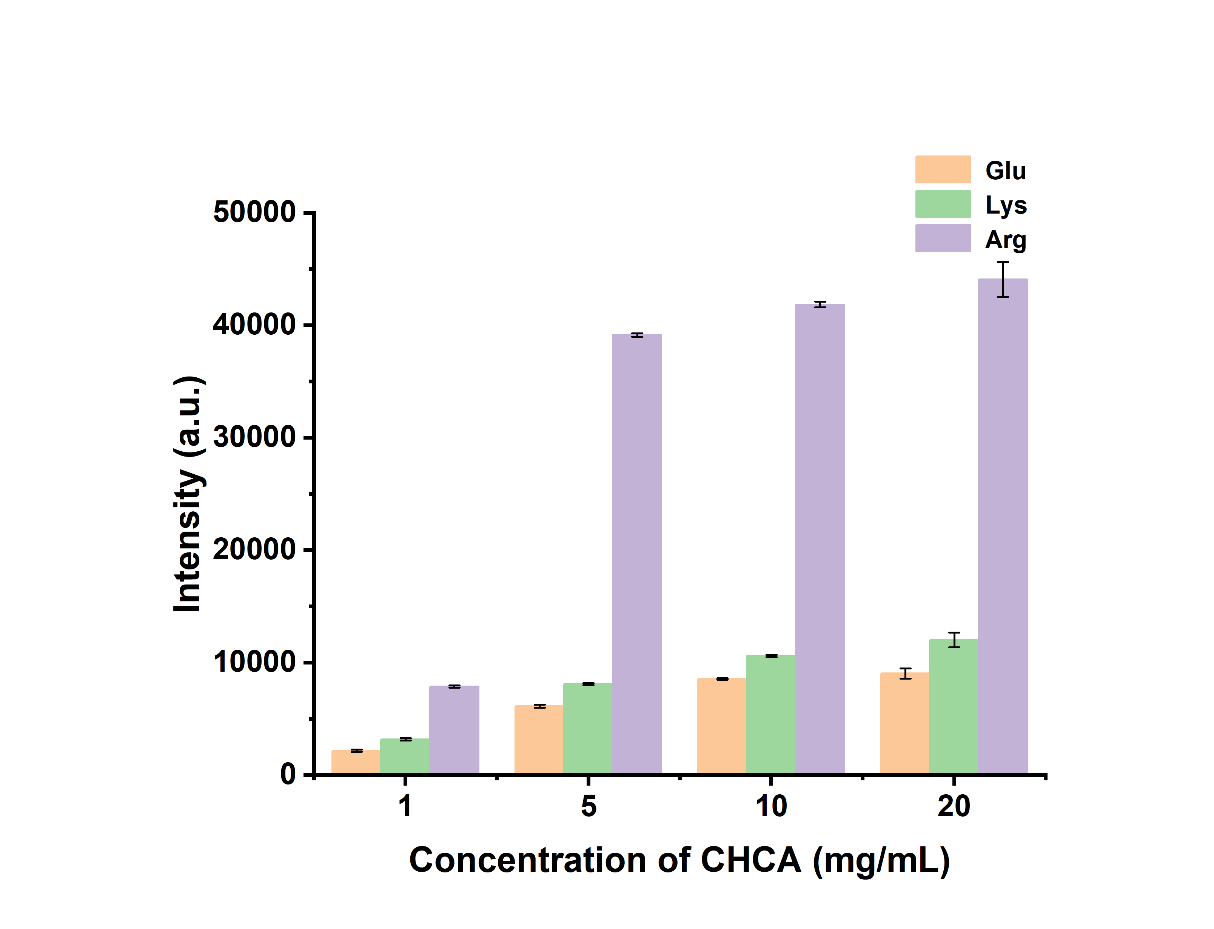


**Fig. S3**. The comparison of signal intensity and the reproducibility of 1mM amino acid standards (Arg, Lys, Glu) detected by different concentrations of CHCA. Error bars indicate standard deviation from triplicate experiments.


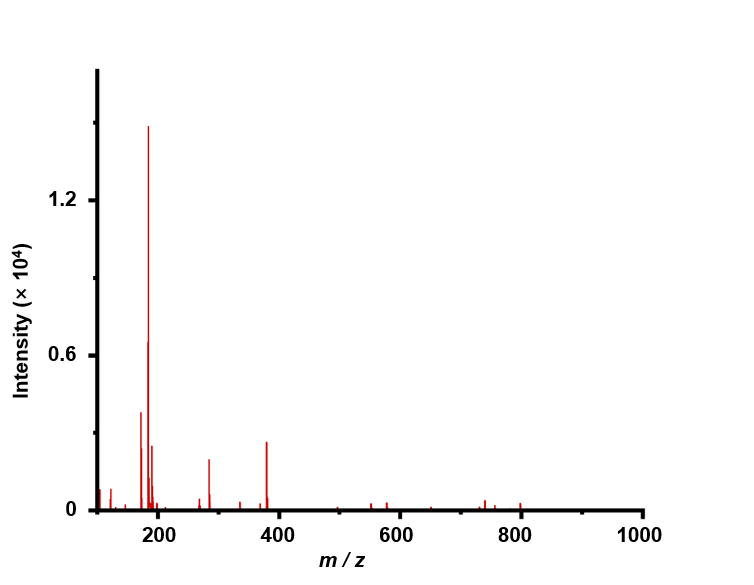


**Fig. S4.** Matrix-assisted laser desorption/Ionization-time of flight mass (MALDI-TOF MS) spectra of EVs model sample based on alpha-cyano-4-hydroxycinnamic acid (CHCA) matrix.


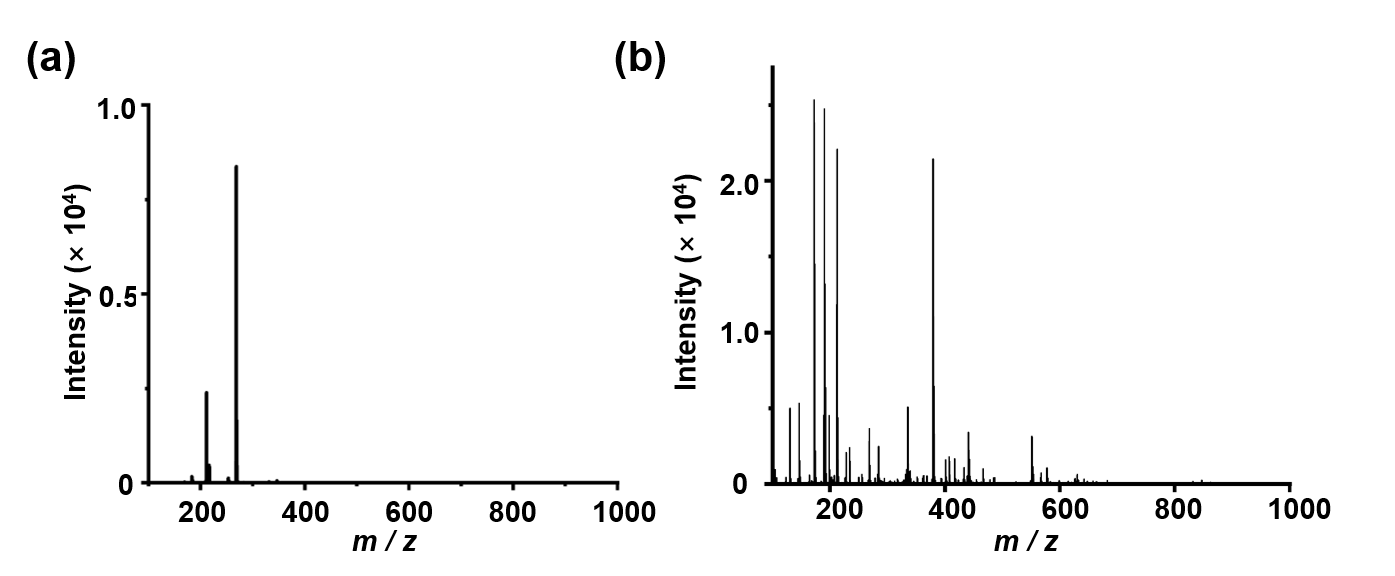


**Fig. S5.** The blank background of (a) Au nanoparticles mixture (AuM) matrix and (b) alpha-cyano-4-hydroxycinnamic acid (CHCA) matrix by matrix-assisted laser desorption/Ionization-time of flight mass spectrometry (MALDI-TOF MS).

**
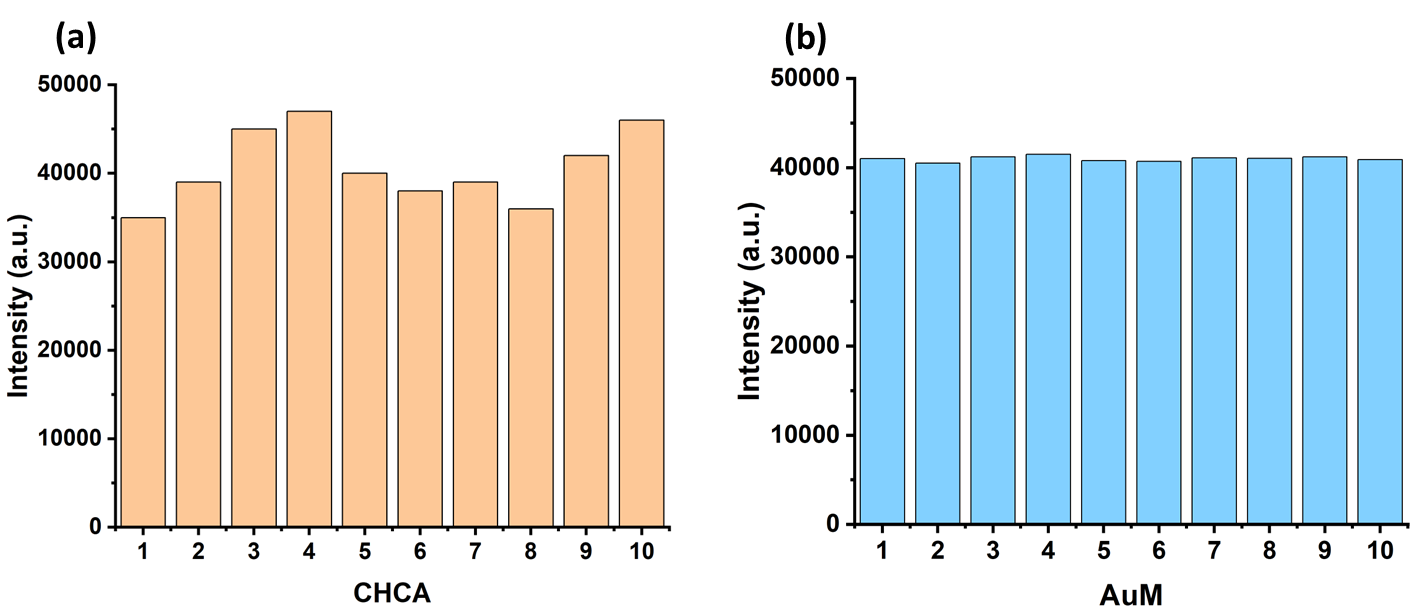
**

**Fig. S6.** The LDI-MS with (a) CHCA and (b) AuM as matrices for the 10 times detection of the Arg (1 mM). The CV (coefficient of variation) values were (a) 18.72% and (b) 1.62%.


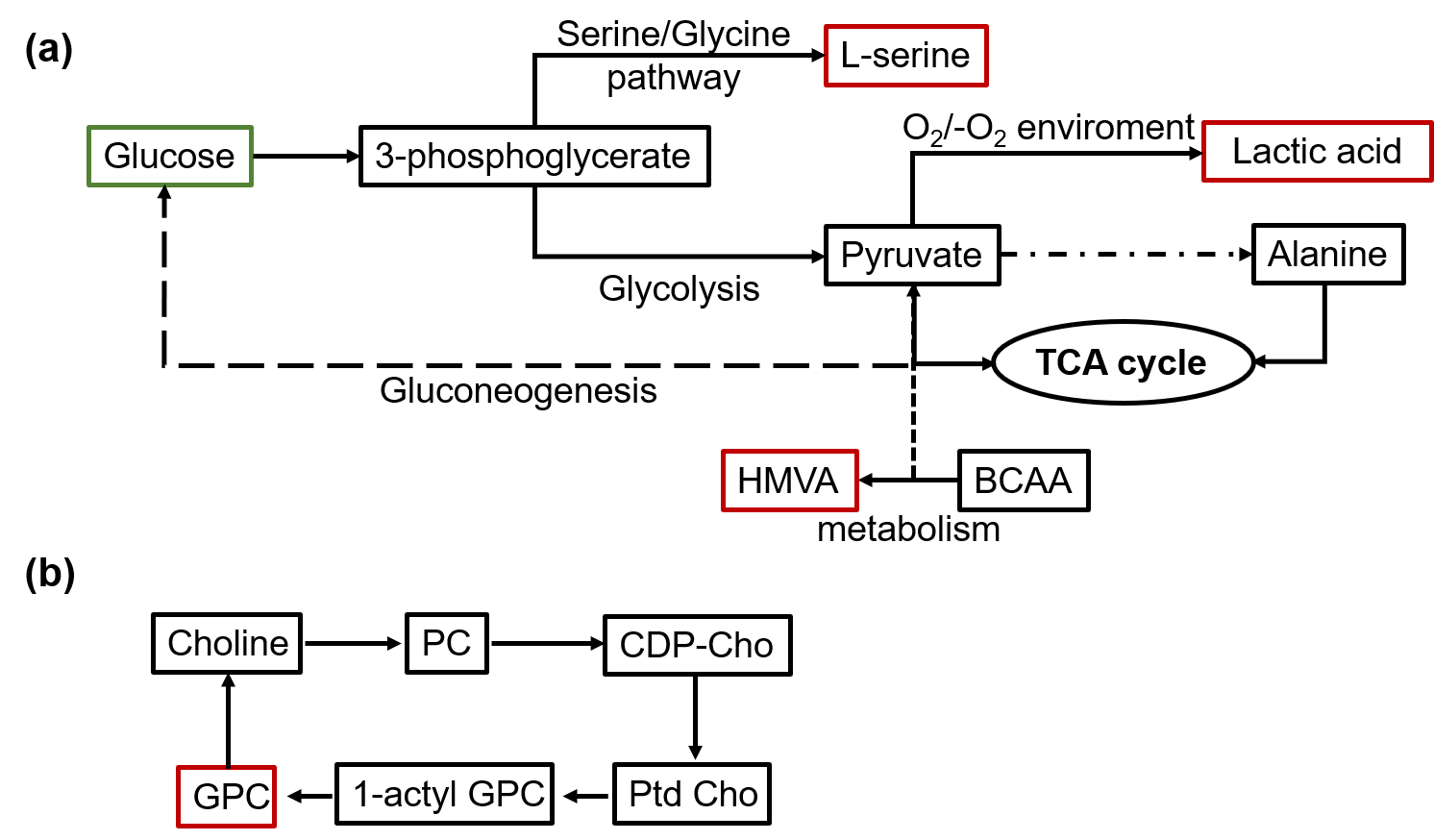


**Fig. S7**. The possible pathway in breast cancer cells. (a) Glucose metabolism, serine/glycine metabolic pathway and branched-chain amino acid (BCAA) metabolic pathway in breast cancer cells. (b) Choline metabolism in breast cancer cells. Red and green represented up-regulated and down-regulated (with fold change (FC) > 1.5 and p-value (*P*) < 0.05) in breast cancer patients compared with healthy donors, respectively.


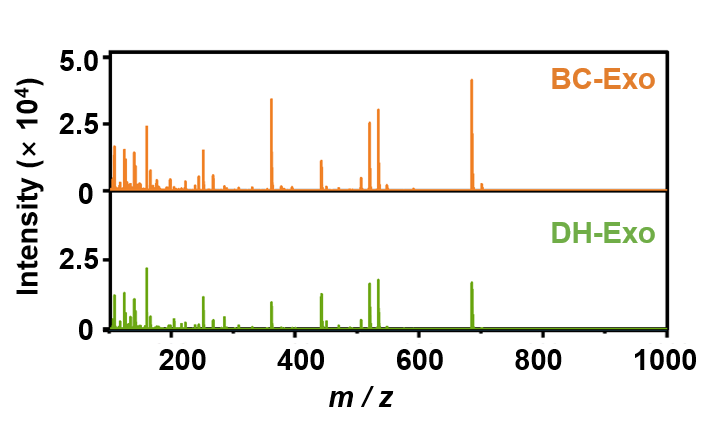


**Fig. S8**. Matrix-assisted laser desorption/Ionization-time of flight mass (MALDI-TOF MS) spectra of BC-EVs and DH-EVs based on Au nanoparticles mixture (AuM) matrix. BC-EVs and DH-EVs represented serum-derived EVs of mice with breast cancer and doxorubicin hydrochloride treatment, respectively.

**
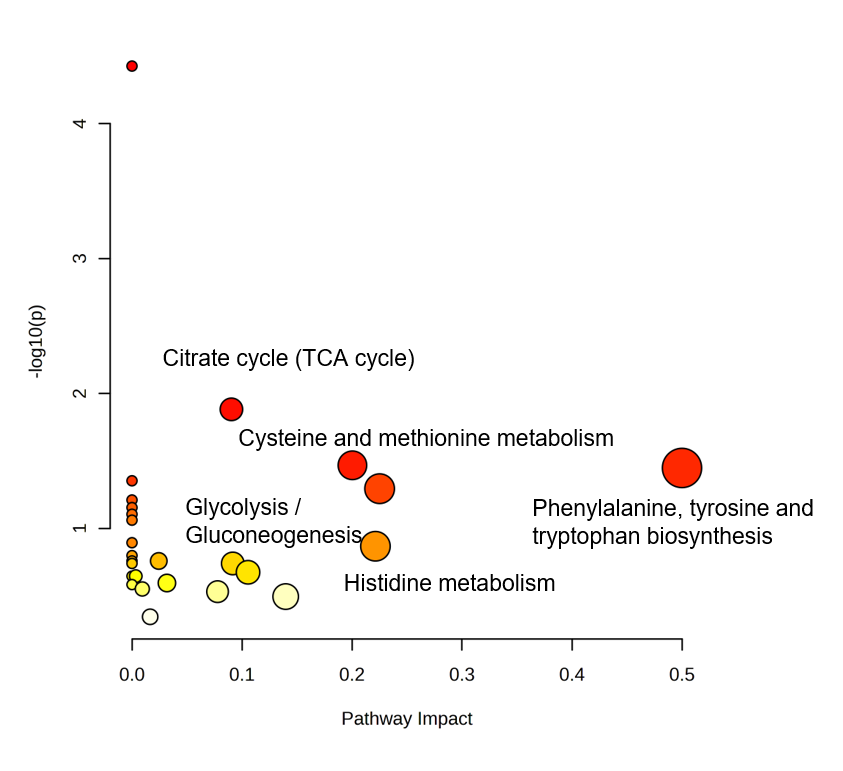
**

**Fig. S9**. Pathway analysis of the key metabolic features in EVs metabolic profiling discrimination of application on cancer treatment monitoring.


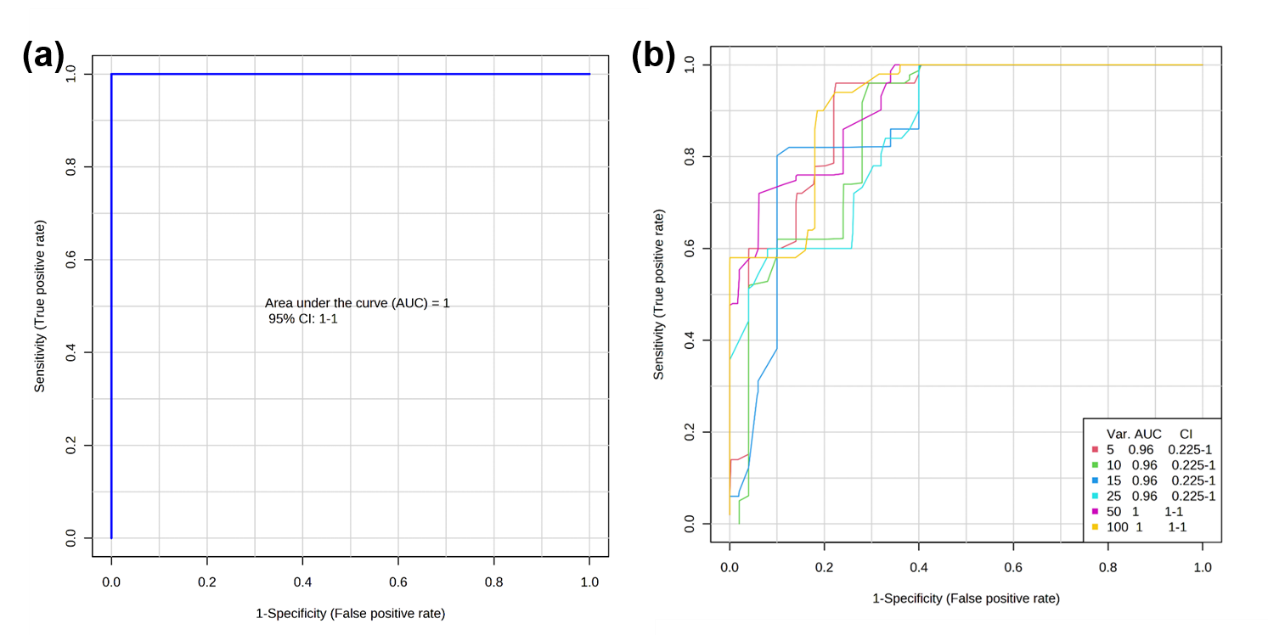


**Fig. S10.** (a) The area under the curve (AUC) value of the classification receiver operating characteristic (ROC) curve for the disease detection panel screened by matrix-assisted laser desorption/Ionization-time of flight mass spectrometry (MALDI-TOF MS) based on Au nanoparticles mixture (AuM) matrix. (b) The AUC value of the ROC curves was 0.96 by using the top 5 features of conventional machine learning method (partial least squares-discriminant analysis, PLS-DA) for classification.

**Table S1**. Significant m/z value of the raw spectrum of model EVs obtained by AuM (AuNS:AuNR=1:3 in volume) -assisted LDI MS.

| No. | *m/z* | No. | *m/z* | No. | *m/z* | No. | *m/z* |
| --- | --- | --- | --- | --- | --- | --- | --- |
| 1 | 112.98 | 2 | 112.05 | 3 | 113.54 | 4 | 115.96 |
| 5 | 122.62 | 6 | 126.94 | 7 | 134.96 | 8 | 135.79 |
| 9 | 144.21 | 10 | 148.80 | 11 | 155.20 | 12 | 160.06 |
| 13 | 171.03 | 14 | 172.11 | 15 | 174.86 | 16 | 177.99 |
| 17 | 178.03 | 18 | 182.03 | 19 | 184.07 | 20 | 190.96 |
| 21 | 203.22 | 22 | 214.86 | 23 | 231.23 | 24 | 237.05 |
| 25 | 242.96 | 26 | 268.27 | 27 | 284.19 | 28 | 340.36 |
| 29 | 380.42 |  |  |  |  |  |  |

**Table S2**. The six potential metabolites serve as biomarkers for breast cancer diagnosis. The terms “up” and “down” represented up-regulated and down-regulated (with fold change (FC) > 1.5 and p-value (*P*) < 0.05) in breast cancer patients compared with healthy donors, respectively.

| Up | *m/z*  (LDI-MS） | HMDB number | Putative metabolite | P value | Fold change | MWD (ppm) | *m/z*  (FT- ICR-MS) | MWD (ppm) |
| --- | --- | --- | --- | --- | --- | --- | --- | --- |
|  | 112.98 | HMDB0001311 | D-Lactic acid | 4.50E-03 | 2.91 | 327 | 112.791 | 68 |
|  | 155.20 | HMDB0000317 | 2-Hydroxy-3-methylpentanoic acid | 9.14E-03 | 2.34 | 235 | 155.172 | 121 |
|  | 280.32 | HMDB0000086 | Glycero-phosphocholine | 9.76E-03 | 1.64 | 201 | 280.251 | 137 |
|  | 144.21 | HMDB0000187 | L-serine | 4.90E-02 | 1.51 | 187 | 144.069 | 28 |
| Down | 178.03 | HMDB0000177 | Histidine | 1.09E-02 | 0.48 | 165 | 178.087 | 59 |
|  | 203.22 | HMDB0000122 | Glucose | 6.95E-03 | 0.59 | 133 | 203.056 | 80 |

(P-values were calculated from peak intensity of detected potential metabolites compared between breast cancer patients and healthy donors; MWD: Molecular Weight Deviation (|(query m/z – molecule adduct m/z)/molecule adduct m/z|*1000000)

**Table S3**. The 13 up-regulated and 11 down-regulated metabolites (with fold change (FC) > 1.5 and p-value (*P*) < 0.05) were identified in the DH-EVs compared with BC-EVs. BC-EVs and DH-EVs represented serum-derived EVs from mice with breast cancer and doxorubicin hydrochloride treatment, respectively.

|  | *m/z*  *(LDI-MS）* | HMDB number | Putative metabolite | P value | Fold change | MWD (ppm) | *m/z*  (FT- ICR-MS) | MWD (ppm) |
| --- | --- | --- | --- | --- | --- | --- | --- | --- |
| Down | 115.96 | HMDB0000162 | Proline | 3.17E-02 | 2.07 | 65 | 116.003 | 79 |
|  | 126.94 | HMDB0000011 | 3-Hydroxybutyric acid | 3.28E-02 | 2.46 | 87 | 127.001 | 63 |
|  | 148.80 | / | / | 4.79E-02 | 1.59 | 90 | 148.812 | 46 |
|  | 172.11 | HMDB0000696 | Methionine | 9.83E-03 | 2.34 | 123 | 172.130 | 78 |
|  | 184.07 | HMDB0001565 | Phosphorylcholine | 1.83E-02 | 11.53 | 154 | 184.119 | 35 |
|  | 214.86 | HMDB0000094 | Citric acid | 9.79E-03 | 7.92 | 169 | 214.965 | 98 |
|  | 231.23 | HMDB0000684 | Kynurenine | 1.03E-02 | 2.18 | 178 | 231.302 | 76 |
|  | 237.05 | HMDB0001031 | Deoxyribose 5-phosphate | 3.52E-02 | 2.07 | 216 | 236.976 | 113 |
|  | 268.27 | HMDB0241651 | 3-Oxobutanoylcarnitine | 2.27E-02 | 5.36 | 283 | 268.291 | 150 |
|  | 284.19 | HMDB0006049 | O-Phosphotyrosine | 5.16E-04 | 2.51 | 315 | 284.311 | 63 |
|  | 340.36 | HMDB0061635 | 3-hydroxynonanoyl carnitine | 4.01E-02 | 3.16 | 356 | 340.371 | 97 |
| Up | 112.05 | HMDB0001310 | D-Alanine | 6.98E-03 | 0.41 | 53 | 111.972 | 156 |
|  | 113.54 | / | / | 4.98E-02 | 0.60 | 64 | 113.670 | 135 |
|  | 122.62 | / | / | 2.66E-02 | 0.64 | 87 | 122.745 | 45 |
|  | 134.96 | HMDB0240714 | Methylphosphonic acid | 1.31E-04 | 0.36 | 107 | 135.004 | 78 |
|  | 135.79 | / | / | 1.59E-03 | 0.41 | 114 | 135.912 | 96 |
|  | 160.06 | HMDB0000574 | L-Cysteine | 3.83E-04 | 0.52 | 160 | 159.928 | 48 |
|  | 171.03 | HMDB0000606 | D-2-Hydroxyglutaric acid | 3.25E-02 | 0.47 | 165 | 170.879 | 36 |
|  | 174.86 | HMDB0000157 | Hypoxanthine | 2.88E-03 | 0.48 | 170 | 174.945 | 29 |
|  | 177.99 | HMDB0000177 | Histidine | 5.61E-03 | 0.61 | 189 | 178.055 | 137 |
|  | 182.03 | HMDB0000158 | L-Tyrosine | 1.75E-02 | 0.49 | 195 | 181.956 | 60 |
|  | 190.96 | HMDB0000263 | Phosphoenolpyruvic acid | 1.50E-02 | 0.66 | 236 | 190.878 | 58 |
|  | 242.96 | HMDB0000472 | 5-Hydroxy-L-tryptophan | 2.38E-03 | 0.51 | 277 | 243.002 | 39 |
|  | 380.42 | HMDB0000277 | Sphingosine 1-phosphate | 4.36E-02 | 0.54 | 328 | 380.501 | 46 |

(P-values were calculated from peak intensity of detected potential metabolites compared between breast cancer mice and doxorubicin hydrochloride treatment mice; MWD: Molecular Weight Deviation (|(query m/z – molecule adduct m/z)/molecule adduct m/z|*1000000)

**Table S4**. The enriched 26 metabolic pathways based on 20 key matched features with specific metabolite structures using Human Metabolome Database (HMDB).

| Pathway name | P value |
| --- | --- |
| Aminoacyl-tRNA biosynthesis | 3.75E-05 |
| Citrate cycle (TCA cycle) | 1.31E-02 |
| Cysteine and methionine metabolism | 3.41E-02 |
| Phenylalanine, tyrosine and tryptophan biosynthesis | 3.57E-02 |
| Synthesis and degradation of ketone bodies | 4.44E-02 |
| Tryptophan metabolism | 5.08E-02 |
| Thiamine metabolism | 6.17E-02 |
| Taurine and hypotaurine metabolism | 7.02E-02 |
| Ubiquinone and other terpenoid-quinone biosynthesis | 7.86E-02 |
| Phenylalanine metabolism | 8.70E-02 |
| Butanoate metabolism | 1.28E-01 |
| Histidine metabolism | 1.36E-01 |
| Pantothenate and CoA biosynthesis | 1.59E-01 |
| beta-Alanine metabolism | 1.75E-01 |
| Sphingolipid metabolism | 1.75E-01 |
| Pyruvate metabolism | 1.82E-01 |
| Pentose phosphate pathway | 1.82E-01 |
| Glycolysis / Gluconeogenesis | 2.12E-01 |
| Alanine, aspartate and glutamate metabolism | 2.26E-01 |
| Glutathione metabolism | 2.26E-01 |
| Glyoxylate and dicarboxylate metabolism | 2.54E-01 |
| Glycine, serine and threonine metabolism | 2.61E-01 |
| Glycerophospholipid metabolism | 2.81E-01 |
| Arginine and proline metabolism | 2.95E-01 |
| Tyrosine metabolism | 3.20E-01 |
| Purine metabolism | 4.52E-01 |
